# Microbial Communities Involved in Methane, Sulfur, and Nitrogen Cycling in the Sediments of the Barents Sea

**DOI:** 10.3390/microorganisms9112362

**Published:** 2021-11-15

**Authors:** Shahjahon Begmatov, Alexander S. Savvichev, Vitaly V. Kadnikov, Alexey V. Beletsky, Igor I. Rusanov, Alexey A. Klyuvitkin, Ekaterina A. Novichkova, Andrey V. Mardanov, Nikolai V. Pimenov, Nikolai V. Ravin

**Affiliations:** 1 Institute of Bioengineering, Research Center of Biotechnology of the Russian Academy of Sciences, 119071 Moscow, Russia; shabegmatov@gmail.com (S.B.); vkadnikov@bk.ru (V.V.K.); mortu@yandex.ru (A.V.B.); mardanov@biengi.ac.ru (A.V.M.); 2 Winogradsky Institute of Microbiology, Research Center of Biotechnology of the Russian Academy of Sciences, 119071 Moscow, Russia; savvichev@mail.ru (A.S.S.); rusanov_igor@mail.ru (I.I.R.); npimenov@mail.ru (N.V.P.); 3 Shirshov Institute of Oceanology of the Russian Academy of Sciences, 117997 Moscow, Russia; klyuvitkin@ocean.ru (A.A.K.); enovichkova@mail.ru (E.A.N.); 4 Il’ichev Pacific Institute of Oceanology, Far East Branch of the Russian Academy of Sciences, 690041 Vladivostok, Russia

**Keywords:** arctic, marine sediments, methane cycle, sulfur cycle, nitrogen cycle, microbial communities, Barents Sea

## Abstract

A combination of physicochemical and radiotracer analysis, high-throughput sequencing of the 16S rRNA, and particulate methane monooxygenase subunit A (*pmoA*) genes was used to link a microbial community profile with methane, sulfur, and nitrogen cycling processes. The objects of study were surface sediments sampled at five stations in the northern part of the Barents Sea. The methane content in the upper layers (0–5 cm) ranged from 0.2 to 2.4 µM and increased with depth (16–19 cm) to 9.5 µM. The rate of methane oxidation in the oxic upper layers varied from 2 to 23 nmol CH_4_ L^−1^ day^−1^ and decreased to 0.3 nmol L^−1^ day^−1^ in the anoxic zone at a depth of 16–19 cm. Sulfate reduction rates were much higher, from 0.3 to 2.8 µmol L^−1^ day^−1^. In the surface sediments, ammonia-oxidizing *Nitrosopumilaceae* were abundant; the subsequent oxidation of nitrite to nitrate can be carried out by *Nitrospira* sp. Aerobic methane oxidation could be performed by uncultured deep-sea cluster 3 of gamma-proteobacterial methanotrophs. Undetectable low levels of methanogenesis were consistent with a near complete absence of methanogens. Anaerobic methane oxidation in the deeper sediments was likely performed by ANME-2a-2b and ANME-2c archaea in consortium with sulfate-reducing *Desulfobacterota*. Sulfide can be oxidized by nitrate-reducing *Sulfurovum* sp. Thus, the sulfur cycle was linked with the anaerobic oxidation of methane and the nitrogen cycle, which included the oxidation of ammonium to nitrate in the oxic zone and denitrification coupled to the oxidation of sulfide in the deeper sediments. Methane concentrations and rates of microbial biogeochemical processes in sediments in the northern part of the Barents Sea were noticeably higher than in oligotrophic areas of the Arctic Ocean, indicating that an increase in methane concentration significantly activates microbial processes.

## 1. Introduction

Microorganisms can influence important biogeochemical cycles in marine ecosystems and play pivotal roles in feedback that magnifies the impacts of global warming in the Arctic region; they are the first responders to the Arctic crisis [1]. Biogeochemical cycles of key biogenic elements (C, S, and N) in Arctic marine ecosystems notably differ from other marine ecosystems. These differences lie in the seasonal variability of all biological processes, the presence of ice sheets, which represent a phase barrier with water columns, and in the occurrence of biological processes in cold environments with negative temperatures. In the Arctic Ocean sediments, at low temperatures, methane can be stored in the form of hydrates, the dissociation of which, in turn, causes the release of methane into the ocean [2,3,4,5,6,7]. 

The Barents Sea is one of the most productive marginal seas in the Arctic, with connections to both the Atlantic and Arctic oceans. Warm and salty Atlantic waters belonging to the northern branch of the Gulf Stream pass through the Norwegian Sea into the open part of the Barents Sea, where they subsequently interact with warm and relatively freshened waters of the coastal Norwegian Current in the south. In the Polar Front zone, the Atlantic water mass meets cold and freshened Arctic waters coming from the Nansen Basin [8,9,10]. Compared with other Arctic seas, the Barents Sea receives a small amount of continental runoff, the main source of which is the Pechora River (70%), which desalinates the southeastern part of the sea [11].

According to the physical and biological ocean models of the Barents Sea [12,13], the average annual gross primary production estimates range from 20 g cm^−2^ yr^−1^ in the seasonally ice-covered northern part to >150 g cm^−2^ yr^−1^ in the southern Barents Sea influenced by Atlantic water [14]. The highest concentration of total organic carbon was found in the marginal ice zone (MIZ; >2% (*w*/*w*)), and the lowest organic carbon concentration was revealed in the southern Barents Sea (<1% (*w*/*w*); [15]. Marine organic matter mainly controls the organic carbon content in the ice-free region. The proximity to the MIZ and transfer of a large number of inorganic and organic substances of terrestrial origin through melting sea ice lead to the fact that organic matter is composed of a mixture of the sea (autochthonous) and terrestrial (allochthonous) sources [15,16,17,18,19]. In turn, a high share of allochthonous organic carbon determines low rates of diagenetic transformation of organic matter and reduces the rates of microbial anaerobic processes. 

Methane is one of the main greenhouse gases. The study of the processes of the methane cycle carried out under strictly anoxic (methanogenesis and anaerobic oxidation of methane) and under oxic (aerobic oxidation of methane) conditions, as well as the study of microbial processes at the border of oxic and anoxic zones of sediments in the Barents Sea provide new insights into the methane cycle in marine sediments. Most of studies dedicated to the methane cycling in the Barents Sea were focused on methane-rich sites, such as methane seeps and mud volcanoes [20,21,22,23]. Particularly, analysis of microbial processes at the Haakon Mosby Mud Volcano revealed that the high methane availability and different fluid flow regimens provide distinct niches for aerobic (*Methylobacter* and *Methylophaga*) and anaerobic (mostly ANME-3 lineage) methanotrophs [22]. Methanotrophic microbial lineages in sediments at cold seep sites (gas hydrate pingos) in the northwestern Barents Sea are quite different and consist of the sole ANME-1 lineage [24]. 

The activity of methane cycling is certainly linked to the biogeochemical cycle of sulfur. Studies of the diversity and distribution of microbial communities involved in sulfur cycling in the Barents Sea illustrated the correlation of rates of anaerobic methane oxidation and sulfide generation, and they revealed thiotrophic microbial mats associated with high fluxes of sulfide [22,25,26]. Nevertheless, the total microbial abundance at the bottom of the Barents Sea may depend on the change of seasons, and it was shown that bacterio- and virioplankton were more abundant in coastal marine areas in late autumn than in winter [27]. This high abundance of microorganisms in the uppermost layers of sediments is linked to the aerobic nature of microbial processes and the availability of reactive organic carbon [28]. 

Most of the above-mentioned studies have focused either on the methane cycle at only a few methane seep sites, or on geomicrobiological processes unrelated to the methane cycle in the sediments of the Barents Sea. The study of microbial communities and methane cycle processes in the sediments can help in understanding the methane cycle processes in the Arctic seas. Here we report the results of studying microbial processes and molecular analysis of microbial communities in the sediments collected at five stations at the northern part of the Barents Sea.

## 2. Materials and Methods

### 2.1. Site Description and Sampling

Sediment samples were collected at five stations at depths from 101 to 1514 m in August 2020 during the 80th cruise of R/V Akademik Mstislav Keldysh [29]. Samples were taken with a multi-corer or Van Veen grab sampler (0.1 m^2^ sampling area, station 6840) into sterile flasks. Sediment samples were represented by aleurite-pelitic silt with various contents of coarse fraction. Three horizons were investigated at station 6841, while at other stations only the surface sediment (0–1 cm) was analyzed (Table 1).

Immediately after lifting the sampler with sediments to the deck, undisturbed sediment samples from each layer of sediment (2.5 cm^3^) were taken into sterile cut-edged plastic syringes and sealed with a butyl rubber septum stopper without air access. All radioisotope measurements were started within two hours after sampling directly in the laboratory of the research vessel.

To analyze the methane content, 2 cm^3^ of the sediment sample was transferred into glass serum vials using a syringe without a needle. About 0.1 g of KOH was added to each vial to stop microbial activity. Seawater, filtered through filters with a pore size of 0.2 μm, was added to a sign marking the volume of the tube’s head space, and the vials were closed with gas-tight butyl rubber stoppers and crimped with aluminum seals. All samples were stored upside down at 4 °C and analyzed for CH_4_ concentrations within 1 month. For molecular genetic studies, syringes with sediment samples were frozen at −18 °C and delivered to the laboratory of the Research Center of Biotechnology of the Russian Academy of Sciences, Moscow.

### 2.2. Chemical Analysis

The pH and eH values in freshly collected sediments were measured with a portable ionometer WTW pH 3110 (Germany) with electrodes WTW Electrode Sen Tix ORP and WTW pH-Electrode Sen Tix 41. The pore water was squeezed out by centrifugation of the sediment samples at 8000 g for 10 min. Alkalinity of the pore water was determined onboard using an alkalinity test (Cat. No. 111090001, Merck, Germany). Concentration of sulfate ion in pore water was determined using a Staier ion chromatograph (Aquilon, Russia) equipped with a conductivity detector and a Dionex IonPac AS22 analytical column, operated isocratically with 4.5 mM NaCO_3_/1.4 mM NaHCO_3_ as eluent at 1.0 mL/min rate at 32 °C.

Methane content in the sediment samples was determined using the headspace method [30]. Methane concentration was measured on a Kristall-2000-M gas chromatograph (Khromatek, Russia) equipped with a flame ionization detector. The detection limit of CH_4_ was 0.1 part-per-million by volume (ppmv) [31]. The molar concentration of methane in sediments was calculated on the basis of those in the flask headspace using Henry’s Law constants [32].

### 2.3. The Total Abundance of Microorganisms

Freshly sampled sediments (0.5 cm^3^) were placed into vials with 14 mL of a 2% glutaraldehyde solution in ultrafiltered seawater and stored at +4 °C. In the laboratory, the volume of the suspension was adjusted to 50 mL with ultrafiltered seawater. The sample was sonicated on a UZV-2/150-TN-RELTEC device (Russia) under the following conditions: sample processing time 4 min, amperage 0.44 A, frequency 15 kHz. After desorption and precipitation of “heavy” particles, 0.5 mL of the suspension was filtered on black polycarbonate filters (Millipore) with a pore diameter of 0.2 µm. Filters were stained with acridine orange solution [33]. The preparations were examined using an epifluorescence microscope (Carl Zeiss, Germany) equipped with an Axio CamHR digital camera at a magnification of × 1000. Cells were counted from a monitor screen in 20 fields of view.

### 2.4. Radiotracer Measurements

The rates of microbial processes of dark CO_2_ assimilation (DCA), sulfate reduction (SR), hydrogenotrophic methanogenesis (MG-h), and methane oxidation (MO) were determined radioisotopically using labeled compounds: NaH^14^CO_3_, specific activity 2.04 GBq mmol^−1^ (Amersham, UK) (5 µCi per sample); ^14^CH_4_, specific activity 1.16 GBq mmol^−1^ (JSC Isotope, Russia) (1 µCi per sample); and Na_2_^35^SO_4_, specific activity 370 mBq mmol^−1^ (Perkin Elmer, Waltham, MA, USA) (5 µCi per sample). Sediment samples (~2.5 cm^3^) were collected into cut-off plastic syringes, preserving the structure of the sediment core, and sealed with gas-tight rubber stoppers to avoid contact of the samples with air. A labeled substrate (0.2 mL as a sterile degassed water solution) was injected through the rubber stopper using a syringe. The samples were incubated for 20 h at in situ temperature (+4 °C). Sediment samples with a preliminarily introduced KOH solution were used as killed controls.

After incubation, the microbial processes (DCA, SR, MG-h, MO) were stopped by injecting 0.5 mL of saturated KOH solution into each experimental sample. After the end of the experiments, the samples were stored at 5–10 °C. Measurement of the radioactivity of the products of microbial activity in both the experimental and control samples was performed as described earlier [34,35]. All experiments were performed in duplicate.

### 2.5. 16S rRNA Amplicon Sequencing and Analysis

The total DNA was extracted from sediment samples using Power Soil DNA isolation kit (MO BIO Laboratories, Inc., Carlsbad, CA, USA). PCR amplification of 16S rRNA gene fragments comprising the V3–V6 variable regions was carried out using the universal prokaryotic primers PRK 341F (5′-CCTAYG GGDBGCWSCAG) and PRK 806R (5′-GGA CTA CNVGGG THTCTAAT) [36]. The PCR fragments were bar-coded using the Nextera XT Index Kit v.2 (Illumina, San Diego, CA, USA) and purified using Agencourt AMPure beads (Beckman Coulter, Brea, CA, USA). The concentrations of PCR products were calculated using the Qubit dsDNA HS Assay Kit (Invitrogen, Carlsbad, CA, USA). All PCR fragments were then mixed and sequenced on Illumina MiSeq (2 × 300 nt from both ends). Pairwise overlapping reads were merged using FLASH v.1.2.11 [37]. The final dataset consisted of 284,185 16S rRNA gene reads (Table 1). 

All sequences were clustered into operational taxonomic units (OTUs) at 97% identity using the USEARCH v. 11 program [38]. Low quality reads and chimeric sequences were removed by the USEARCH algorithms. To calculate OTU abundances, all reads obtained for a given sample (including singleton and low-quality reads) were mapped to OTU sequences at a 97% global identity threshold by Usearch. The taxonomic assignment of OTUs was performed by searching against the SILVA v.138 rRNA sequence database using the VSEARCH v. 2.14.1 algorithm [39]. OTUs assigned to chloroplasts, mitochondria, and eukaryotes, as well as OTUs containing only one read in the entire dataset and likely resulting from sequencing errors, were excluded from the analysis.

The Chao1 and Shannon diversity indices at a 97% OTU cut-off level were calculated using Usearch v.11 [38]. To avoid sequencing depth bias, the number of reads generated for each sample were randomly sub-sampled to the size of the smallest set (reads from 6840 sample) using the “otutab_rare” command of Usearch.

### 2.6. Sequencing and Analysis of pmoA Gene Sequences

The DNA fragments encoding the *pmoA* (particulate methane monooxygenase subunit A) gene were amplified using PCR with the primers A189b (5′-GGNGACTGGGACTTYTGG) and A682 (5′-GAASGCNGAGAAGAASGC) [40]. The following program was used: 96 °C for 2 min, followed by 40 cycles of 96 °C for 30 s, 57 °C for 30 s, and 72 °C for 40 s, and a final elongation at 72 °C for 10 min. The PCR fragments were sequenced on Illumina MiSeq (2 × 300 nt from both ends). A total of 17,352, 10,749, 4169, 1766, 6079, 5140, and 5434 of sequences were obtained for the samples 0–1 cm (6840 station), 0–5 cm (6841), 6–7 cm (6841), 16–19 cm (6841), 0–1 cm (6844), 0–1 cm (6849), and 0–1 cm (6864), respectively.

Clustering of the nucleotide sequences of *pmoA* was carried out at a 97% identity threshold in the same manner as for the 16S rRNA gene sequences. The obtained OTUs were taxonomically and functionally assigned using BLASTP searches of the deduced amino acid sequences against the NCBI non-redundant protein sequence database. 

For phylogenetic analysis, the deduced amino acid sequences of *pmoA* OTUs and PmoA sequences of known methanotrophic lineages were aligned by the Muscle algorithm using MEGA7 [41]. The maximum likelihood phylogenetic tree was constructed using PhyML v. 3.3 with default parameters [42].

## 3. Results

### 3.1. Characterization of the Sampling Sites and Microbial Processes

**Figure 1:**
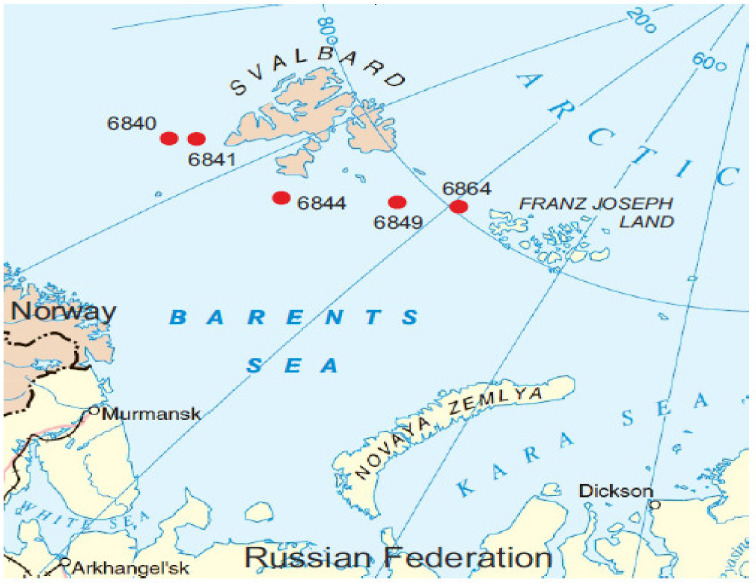
Location of sampling stations.

Stations 6840, 6841, and 6844 were located south of the Svalbard (Spitsbergen) archipelago, while stations 6849 and 6864 were in the northernmost part of the Barents Sea, between Svalbard and Franz Josef Land archipelagos (Figure 1). The stations were located on the shelf except for station 6840, which was located on the continental slope. 

Continental slope sediments (station 6840) sampled from a depth of about 1500 m in the near-surface layer (0–8 cm) were represented by olive brown aleurite-pelitic silt with an admixture of sandy material (~5–10%). The sandy material contained foraminifera shells and volcanic glass.

Station 6841 was located at the Sturfjord trough, one of the known methane seep areas [43,44], in the zone of the most pronounced torch reaching a height of more than 100 m above the bottom level (80th AMK cruise report, 2020). In the 0–2 cm horizon a silty-pelitic silt of dark olive-brown color was exposed, then along the core it turned into aleurite-pelitic silt of dark olive-gray and deep black-gray colors with a large amount of hydrotroilite smears, spots, and micro-layers. Methane-associated authigenic carbonate crusts were found at depths of about 2 cm and below (especially in the 14–22 cm horizon). The size of the crusts varied from 0.5 to 4.5 cm. 

The other sediment samples were collected along a transect from the Sturfjord to the Franz Victoria Trough (stations 6844, 6849, 6864). The sediments were represented by strongly bioturbated silty dark yellowish-brown pelitic silts, oxidized at depths of up to 2 cm. An increase in the coarse fraction was noted at station 6864. 

The surface sediments (0–1 cm) consisted of oxidized aleurite-pelitic silt (Eh between +100 and +125 mV) with the fluffy layer. The deeper sediments collected at station 6841 were anoxic, Eh varied from −80 to −120 mV. The pH values corresponded to the characteristics of seawater and did not change with depth, while alkalinity considerably increased (Table 2).

The previous investigations in the Arctic seas showed that coarsening of the particulates that compose the fluffy layer was related not only to the physicochemical processes but also to the formation of the organomineral particles at the expense of the increase of the organic carbon, including the biomass of the microorganisms, in the fluffy layer as compared with the particulates in the suprabottom water [45]. In the studied sediments, fluffy layer was most pronounced at station 6841, where it was represented by flakes of a dark grayish-brown color.

Assessment of total microbial abundance carried out by the microscopy of stained samples revealed similar values at different stations and the expected decrease in the concentration of microorganisms with depth. The rate of dark carbon assimilation, which reflected an integral metabolic activity of microorganisms, decreased with depth of sediments much more strongly than the total microbial count, which indicates a lower metabolic activity of microorganisms in the deeper anaerobic zone (Table 2).

Methane concentration in the surface sediments (0–1 cm layer) at all stations except for 6841 was relatively low, varying from 0.2 to 0.6 µmol L^−1^. At station 6841, located in the methane seep area, the methane content was several times higher and increased with depth reaching 9.5 µmol L^−1^ in the deepest analyzed horizon (16–19 cm). Such values of methane concentration are typical for the peripheral zones of methane seeps. In the zone of active seepage, the methane concentration in surface sediments can reach tens and even hundreds µmol L^−1^ [25,46].

The rates of metanogenesis were below the reliable detection limit of 2 nmol L^−1^ day^−1^ in all samples, but these values could be underestimated since they were measured only for hydrogenotrophic methanogenesis with H_2_/CO_2_ as a substrate. Data on the rates of methane oxidation (MO) at methane-rich station 6841, where not only the surface sediments but also two deeper anoxic sediment layers were analyzed, showed that below the oxidized zone activity of methanotrophs decreased drastically, indicating that MO mostly proceeded aerobically. 

The concentration of sulfate in all samples was comparable with that in seawater (about 28 mM). At station 6841 a slight decrease in the content of sulfates was observed when deepening into the sedimentary strata. The sulfate reduction rates were very high in almost all sediment samples (1–3 µmol L^−1^ day^−1^), comparable with the rates of dark carbon assimilation (Table 2). Active sulfate reduction in the upper layers of the sediment indicated the presence of anaerobic micro-niches. The rate of sulfate reduction was much lower at station 6864 where the sediment was taken as fluff-like substance and was probably well oxygenated.

### 3.2. Diversity and Composition of Microbial Communities

**Figure 2:**
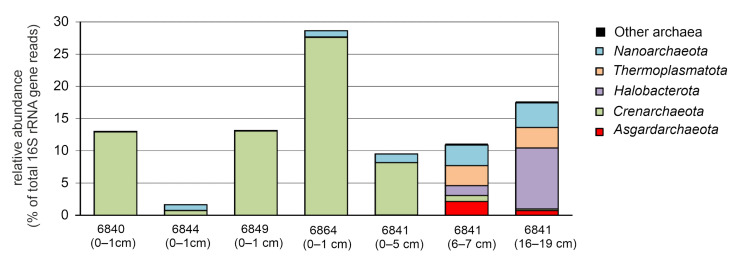
Relative abundancies of taxonomic groups of Archaea according to 16S rRNA gene profiling.

**Figure 3:**
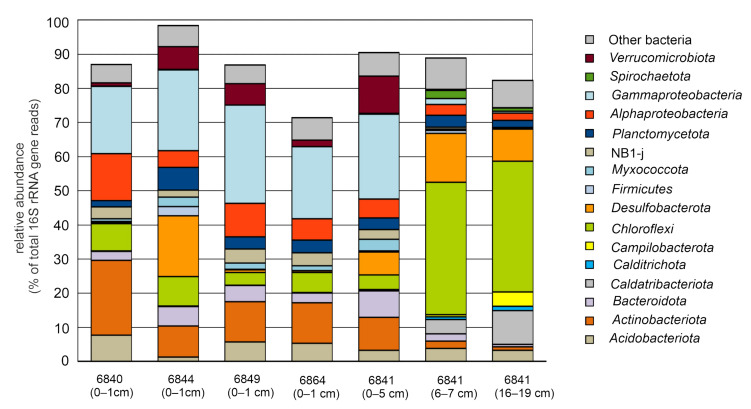
Relative abundancies of taxonomic groups of bacteria according to 16S rRNA gene profiling.

To characterize the compositions of microbial communities, a total of 284,185 sequences of 16S rRNA gene fragments were determined. As a result of clustering the obtained sequences, 4646 bacterial and 551 archaeal OTUs were identified at the level of 97% sequence identity. Alpha diversity indices indicated high bacterial and much lower archaeal diversity in all sediment samples. Pronounced decrease in bacterial diversity and richness was observed in the deep sediment sample (16–19 cm) (Table 3). The results of the taxonomic classification of the OTUs are shown in Figure 2 and Figure 3, and in Supplementary Table S1.

Archaea accounted for 1.7 to 28.6% of all 16S rRNA gene sequences. The overall abundance of archaea did not correlate with the sediment depth since the minimum and maximum values were observed in the upper sediment layers at stations 6844 and 6864, respectively. Archaea were represented by nine phyla defined in genome-based taxonomic system [47]—*Aenigmarchaeota, Asgardarchaeota, Altiarchaeota, Crenarchaeota, Euryarchaeota, Hadarchaeota, Halobacterota, Nanoarchaeota,* and *Thermoplasmatota*. Members of these phyla were unevenly distributed between the upper oxic layer and lower anoxic layers of sediments (Figure 2). 

*Asgardarchaeota*, assigned to *Heimdallarchaeia*, *Lokiarchaeia*, and *Odinarchaeia* were found only in anoxic sediments at station 6841 and in minor amounts (<2.15% of all 16S rRNA sequences). Likewise, Marine Benthic Group D (phylum *Thermoplasmatota*) was found only in deep layers (6–7 and 16–19 cm) and constituted 2.0% and 2.9% of the community. A similar distribution pattern was also observed for less numerous *Aenigmarchaeota*, *Bathyarchaeia* (phylum *Crenarchaeota*), and *Hadarchaeota*. Notably, two lineages of anaerobic methane-oxidizing archaea (ANME)—ANME-2a-2b and ANME-2c (phylum *Halobacterota*)—were found only in anoxic sediments at station 6841 and were most abundant in the deepest layer (9.47%). Members of archaeal phylum *Nanoarchaeota* were found in the all horizons and mostly accumulated in the anoxic sediments (up to 3.9% of reads). The opposite distribution pattern was observed for the *Crenarchaeota* assigned to the family *Nitrosopumilaceae*, comprising aerobic ammonia-oxidizing archaea [48]. *Nitrosopumilaceae* was one of the most numerous microbial lineages (from 8.1% to 27.6%) in all upper sediments, except for the sample from station 6844. Another notable finding was the near complete absence of methanogens, among which only probable methylotrophic methanogens of the candidate order *Methanofastidiosales* were found, but their relative abundance was less than 0.05%.

The domain Bacteria was represented by 20 phyla, accounting for more 1% of 16S rRNA sequences in at least one sample (Figure 3 and Supplementary Table S1). The most abundant phylum, *Proteobacteria*, mostly of classes alpha and gamma, formed about one third of communities in surface sediments and were less abundant in the deep layers. Among alpha-proteobacteria, the family *Hyphomicrobiaceae* was the most abundant (up to 4% of 16S rRNA reads) and occurred in both surface and deep sediments. The second major lineage of alpha-proteobacteria, *Kiloniellaceae*, predominantly was found in surface sediments. Gamma-proteobacteria clearly prevailed in the upper sediment samples (20–29% versus <2% in the deep sediments) and were mostly composed of taxa unclassified even at the family level. Among the cultured lineages were found members of the families *Colwelliaceae* (*Colwellia* sp.), *Coxiellaceae* (*Coxiella* sp.), *Psychromonadaceae* (*Psychromonas* sp.), *Shewanellaceae* (*Psychrobium* sp.), *Nitrosomonadaceae* (*Nitrosomonas* sp.), *Halieaceae* (*Halioglobus* sp.), *Spongiibacteraceae*, *Nitrosococcaceae, Woeseiaceae* (*Woeseia* sp.), *Thiotrichaceae,* and *Thiohalorhabdaceae*. 

*Chloroflexi* dominated in the deep sediments at station 6841, accumulating nearly 40% of sequences, but it was numerous also in near-surface sediments samples. Most of *Chloroflexi* OTUs were assigned to classes *Anaerolineae* and *Dehalococcoidia*, while *Chloroflexia* and members of uncultured candidate classes JG30-KF-CM66, TK17, and KD4–96 were found in minor amounts. The majority of *Anaerolineae* OTUs were placed in the family *Anaerolineaceae* but were not classified at the genus level. Members of the candidate order SBR1031 were relatively numerous only in the deep sediments (1.7–3.0%). Analysis of the relative abundance of particular *Anaerolineae* OTUs in different samples revealed a clear niche specialization: some OTUs were present in the surface layers, while others were present in the deep sediments (Supplementary Table S1). Contrary to *Anaerolineae*, most of *Dehalococcoidia* OTUs represented uncultured order-level divisions phylogenetically distant from known isolates. Members of the order S085 were found in the surface sediments, while GIF3, GIF9, Napoli-4B-65, and MSBL5 were prevalent in the deep layers.

The phylum *Verrucomicrobia* was mostly represented by the family *Rubritaleaceae* (genera *Haloferula, Luteolibacter, Persicirhabdus, Roseibacillus*, and *Rubritalea*) found in the surface sediments (up to 8%). A similar distribution was observed for *Actinobacteria* (mostly of the order *Actinomarinales*), whose share reached 18.7%. Members of *Planctomycetes* (*Phycisphaerales*), *Nitrospirota* (*Nitrospira* sp.), *Myxococcota*, *Firmicutes*, *Bacteroidetes* (*Cyclobacteriaceae*), *Gemmatimonadota, Patescibacteria, Latescibacterota,* subgroups 21 and 22 of *Acidobacteria*, and candidate division NB1-j were also found preferentially in the upper sediments, while members of *Acetothermia* and *Spirochaetota* were predominant in the deep layers.

Bacteria of the phylum *Desulfobacterota* (delta-proteobacteria in traditional taxonomy) were abundant in the upper sediments at station 6844 and in all sediment samples collected at station 6841, but they were found in minor amounts in upper sediments at stations 6840, 6849, and 6864. Interestingly, in the upper sediments at stations 6841 and 6844, most of *Desulfobacterota* belonged to the families *Desulfobulbaceae, Desulfocapsaceae*, and Sva1033 group, while in the deep sediments (station 6841, 6–7 cm and 16–19 cm layers) members of *Desulfatiglandaceae* (*Desulfatiglans* sp.), *Desulfosarcinaceae*, *Dissulfuribacteraceae* (SEEP-SRB2 group), and *Syntrophobacterales* prevailed.

Several bacterial lineages were found preferentially in deep sediments and were nearly absent in the surface sediments samples. The most abundant was the phylum *Caldatribacteriota* (JS1 lineage), representing 4.2% and 9.9% of the microbiome in the 6–7 cm and 16–19 cm horizons, respectively. *Sulfurovum* sp. (phylum *Campilobacterota*) and members of the phylum *Calditrichota* were also found mostly in the deep sediments, and their shares increased with depth. *Aminicenantes* (recognized as class *Aminicenantia* in the phylum *Acidobacteria* in the GTDB taxonomy) were found exclusively in the deep sediments, where they accounted for ~3% of 16S rRNA sequences.

In addition to the major bacterial lineages, 36 other phylum-level divisions were identified—namely, *Aerophobota, Armatimonadota, Bdellovibrionota, Cloacimonadota, Cyanobacteria, Dadabacteria, Deferrisomatota, Deinococcota, Dependentiae, Elusimicrobiota, Entotheonellaeota, Fermentibacterota, Fusobacteriota, Fibrobacterota, Hydrogenedentes, Margulisbacteria, Methylomirabilota, Modulibacteria, Nitrospinota, Nitrospirota, Schekmanbacteria, Sumerlaeota, Zixibacteria*, 10bav-F6, BHI80-139, CK-2C2-2, FCPU426, LCP-89, SAR406_clade, MBNT15, NKB15, RCP2-54, SAR324_clade, TA06, WOR-1, and WS2. Their shares were less than 1% in all analyzed samples.

### 3.3. Aerobic Methanotrophs Revealed by pmoA Gene Profiling

To identify methanotrophic bacterial lineages, we amplified and sequenced the libraries targeting the genes coding for a conserved region of the particulate methane monooxygenase subunit A (*pmoA*). The *pmoA* genes can be used in the reconstruction of phylogenetic relationships among aerobic methanotrophs (reviewed in [49]). 

Taxonomic assignment of obtained OTUs showed that only two of them (OTU9 and OTU32) represented *pmoA* genes, and six OTUs belonged to evolutionary related ammonia monooxygenase (*amoA*), which is likely a consequence of co-amplification. All *amoA* OTUs were assigned to the genus *Nitrosomonas*, also identified by 16S rRNA gene profiling. The *pmoA* OTU9 was detected only in the deep sediments (6–7 cm) of station 6841, while OTU32 occurred additionally in the upper sampled horizons at stations 6841, 6844, and 6849.

**Figure 4:**
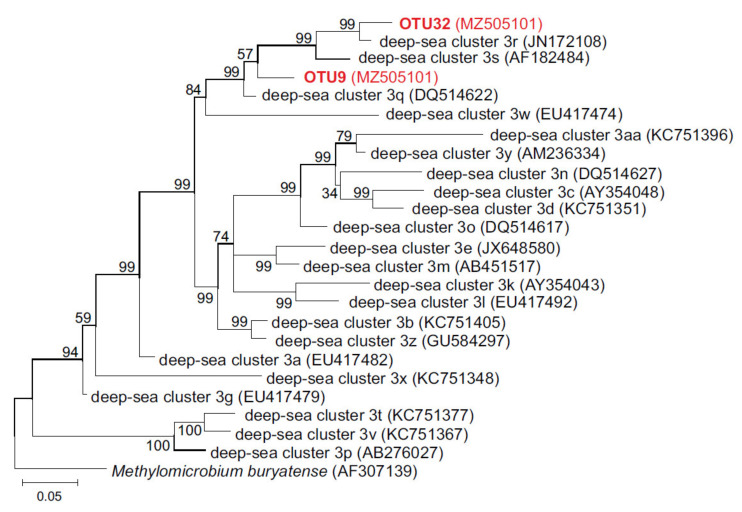
Phylogenetic tree based on the deduced amino acid sequences of *pmoA* OTUs and representatives of deep-sea cluster 3 [49]. OTUs found in this work are shown in red. The support values for the internal nodes were estimated by approximate Bayes test in PhyML. GenBank accession numbers are shown in parentheses. *pmoA* of *Methylomicrobium buryatense* was used to root the tree.

The search against GenBank revealed that OTU9 had 97.45% nucleotide sequence identity to *pmoA* sequence DQ514622 assigned to deep-sea cluster 3q [49], while OTU32 was closely related (95.65% identity) to sequence JN172108 from deep-sea cluster 3r [49]. The two *pmoA* OTUs were 86.12% identical. Taking into account the proposed cut-off values at 10% and 17% *pmoA* sequence dissimilarity for species and genus delineation [50], identified OTUs probably represented different species of the same genus, belonging to uncultured deep-sea cluster 3 of type 1a methanotrophs [49]. Phylogenetic analysis of deduced amino acid sequences for *pmoA* OTUs also confirmed their affiliation with deep-sea cluster 3 (Figure 4).

## 4. Discussion

### 4.1. Methane Cycle

Microbial communities of sediments of the Arctic seas are actively studied using molecular genetic approaches [51,52,53,54]; significantly fewer studies analyze the rates of microbial processes. In this work, we characterized the microbial communities of the surface layers of sediments in the northern part of the Barents Sea and characterized the rates of most important biogeochemical processes associated with carbon and sulfur cycles.

Methane is an end product of microbial decomposition of organic matter under anaerobic conditions and can accumulate in significant amounts in sediments of both fresh and marine water bodies [55]. Methane can accumulate in deep sediments in the form of gas hydrates and be released on the seabed as methane seeps. However, methane concentrations in the upper layers of sediments at most stations did not exceed 1 μM, and only at station 6841 it was several times higher (2.4 μM). Most of the autochthonous organic matter reaching the bottom appeared to be oxidized in the upper layers of sediments, as indicated by the high rate of carbon assimilation and abundance of aerobic heterotrophic bacteria (*Acidobacteria, Bacteriodetes, Verrucomicrobia,* alpha- and gamma-proteobacteria). In deeper horizons sampled at station 6841, the concentration of methane increased by more than an order of magnitude. However, the low rate of methanogenesis and the near absence of methanogens in microbial communities even in anoxic sediments indicated that methane was not formed here but that it migrated from deeper layers to the surface, where its aerobic and anaerobic oxidation occurred [56]. Probably, methanogenesis in the studied sediments was outcompeted by active sulfate reduction [57], and the sulfate–methane transition zone was located deeper than the studied sediment horizon.

The anaerobic oxidation of methane (AOM) is a crucial sink of methane in anoxic environments. AOM coupled to the reduction of sulfate could be carried out by anaerobic methane-oxidizing archaea (ANME) [58,59]. Both active methane oxidation and ANME archaea were found in anoxic sediments at station 6841. The relative abundance of ANME increased with depth. Among known ANME lineages ANME-2a-2b and ANME-2c clades were found. The 6–7 cm layer was dominated by ANME-2a-2b, while the ANME-2c clade was found mostly in the deepest layer (16–19 cm). In marine sediments, ANME clades are usually distributed by zone: ANME 2a-2b dominates upper layers, while ANME-2c and/or ANME-1 abundance increases in deeper zones. This zonation indicates ecological niche separation [60].

In anoxic sediments collected at station 6841 in addition to ANME archaea, sulfate-reducing delta-proteobacteria (phylum *Desulfobacterota* in genome –based taxonomy) were found. In the 6–7 cm layer among sulfate reducers, representatives of *Desulfosarcinaceae* (SEEP-SRB1 group) and *Desulfatiglandaceae* (genus *Desulfatiglans*) prevailed, and in the 16–19 cm layer, *Dissulfuribacteraceae* (SEEP-SRB2 group) and *Desulfatiglandaceae* were most numerous, while the share of *Desulfosarcinaceae* was much lower. Some of these groups are known as partners of the ANME archaea. The common sulfate-reducing bacteria that are usually associated with ANME belong to the *Desulfosarcina*/*Desulfococcus* clade [61,62]. Co-occurrence of ANME-2a-2b and SEEP-SRB1 group is consistent with data showing that AOM is associated with sulfate reduction in an enrichment culture of ANME-2a/b and SEEP-SRB1 sulfate reducers [63,64]. Likewise, SEEP-SRB2 members occurred in association with ANME-2 archaea [65,66]. ANME-2c subgroup was found to be in association with the seepSRB2, seepSRB1a, and seepDBB group of the *Desulfobulbaceae* [65,67].

Methane oxidation rates in the upper layers of sediments (0–1 cm) were several times higher than in deep anoxic layers, while ANME archaea were absent (Table 2, Figure 2). Another group of anaerobic methanotrophs, nitrite-reducing bacteria of the family *Methylomirabilaceae* [68], were found only at stations 6844 and 6849 in minor amounts (~ 0.3%). This indicates that the oxidation of methane in the upper layers is mostly carried out aerobically. However, the known cultivated species of aerobic methanotrophs were not revealed by 16S rRNA gene profiling. Methane oxidation can be carried out by methylotrophs that could utilize C1 substrates as a sole source of energy and carbon [69]. Methylotrophs often coexist with methanotrophs and can contribute to the methane oxidation process [70]. Methylotrophs were found among cultivated species of the family *Hyphomicrobiaceae* (alpha-proteobacteria), the share of which in sediments was up to 4%. *Hyphomicrobium vulgare* can utilize methanol and engage in synergistic interactions with methanotrophs [71]. It is assumed that some members of *Hyphomicrobiaceae* can oxidize methane. Analysis of methanotroph genomes from permafrost soils revealed two novel genomes of potential methanotrophic *Hyphomicrobiaceae* [72]. Members of the family *Methyloligellaceae* detected in all sediment samples can utilize both methylated compound and methane [70]. Particularly, *Methyloceanibacter* strain R-67174, isolated from North Sea sediments, was capable of oxidizing methane as a sole source of carbon and energy [73].

Some representatives of uncultured lineages of gamma-proteobacteria, which were numerous in the upper layers of sediments and accounted for up to one third of microbial communities, can also be methanotrophs. The finding, as a result of sequencing the *pmoA* gene library, of two OTUs assigned to deep-sea cluster 3, is consistent with this assumption. This cluster represented uncultured members of the family *Methylococcaceae* and was identified nearly exclusively in marine habitats [49].

Interestingly, 16S rRNA sequences assigned to the genus *Nitrosomonas*, as well corresponding *amoA* gene sequences, were found in all upper sediment samples. Moreover, *amo* and *pmo* are evolutionary related enzymes [74], and it was shown long ago that ammonia-oxidizing bacteria such as *Nitrosomonas* sp. can oxidize methane to methanol via the nonspecific action of the ammonia monooxygenase [75,76]. Although efficiency of methane oxidation by *Nitrosomonas* is much lower than by true methanotrophs, the high-yield production of methanol from methane by ammonia-oxidizing bacteria (AOB) is feasible [77]. Therefore, *Nitrosomonas* sp. could contribute to methane oxidation in the upper layer of sediments of the Barents Sea. Indirectly, this is indicated by the presence of *Hyphomicrobiaceae* methylotrophs, capable of completing this process by oxidizing methanol produced by AOB. 

### 4.2. Sulfur Cycle

The concentration of sulfate in all sediment samples approximately corresponded to its content in seawater, and the intensity of sulfate reduction was comparable with the intensity of carbon assimilation and exceeded the rate of methane oxidation by several orders of magnitude. The abundance of sulphate-reducing microorganisms is usually low in the uppermost oxygenated layers of sediments, while in the underlying anoxic zones it reaches a maximum and then decreases by depth and age of sediments into the sulphate-depleted methane zone [78]. 

Among known sulfate-reducing prokaryotes, only delta-proteobacteria were found. In the upper layers of sediments, the share of sulfate reducers was low (except for station 6844). At station 6841, it increased to 17.39% at a depth of 6–7 cm, and at a depth of 16–19 cm it was 12.9%. Probably, the sulfate-depleted methane-rich zone was located deeper.

In addition to the above-mentioned sulfate-reducing partners of ANME archaea, the presence of the family *Desulfobulbaceae* was notable. This group was abundant only in the upper sediments at stations 6841 and 6844, accounting for 2.0% and 5.5% of 16S rRNA reads, respectively. *Desulfobulbaceae* are metabolically diverse bacteria capable of dissimilatory iron reduction [79], oxidation of elemental sulfur [80], and sulfate and sulfite reduction in the complete oxidation of organic matter [81]. Cable bacteria of the genus *Candidatus* Electrothrix (the family *Desulfobulbaceae)* forms filaments transferring electrons between the sulfidic and oxic zone up to centimeter distance. They are not capable of performing dissimilatory sulfate reduction; instead, in the sulfidic zone, they oxidize sulfide (H_2_S) using oxygen or nitrate as an electron acceptor [78,82,83]. Three OTUs of the family *Desulfobulbaceae* were assigned to *Ca*. Electrothrix, but their share in the communities did not exceed 0.5%. The search in the GenBank for sequences related to the most abundant *Desulfobulbaceae* OTU, whose share was 1.7% in sample 6841 (0–5 cm layer) and 4.3% in sample 6844, showed that the closest hit was *Ca*. Electrothrix communis, but the sequence identity was only 92.75%. It is possible that the identified members of *Desulfobulbaceae* carry out the transfer of electrons between the aerobic and anaerobic layers of the sediments. This hypothesis is consistent with their absence in deeper anoxic sediments.

Like *Desulfobulbaceae*, the uncultivated Sva1033 sediment group of *Desulfobacterota* was relatively abundant (up to 7.5%) only in the upper layers of sediments of stations 6841 and 6844. Although the metabolic potential of Sva1033 remains unknown, it was hypothesized that like *Desulfobulbus* they could perform metal and/or sulfate reduction in Arctic fjord sediments [84].

The product of sulfate reduction, hydrogen sulfide, as well as other reduced sulfur compounds and elemental sulfur, can be oxidized by sulfur-oxidizing bacteria (SOB) [85,86,87,88]. Two known lineages of SOB were identified. The first was gamma-proteobacteria of the family *Thiohalorhabdaceae* present in the upper sediment in minor amounts (<0.5%). A cultivated member of this family, *Thiohalorhabdus denitrificans,* is a chemolithoautotrophic bacterium using thiosulfate and tetrathionate as electron donors and nitrate as electron acceptor [89]. The second, more numerous group of SOB, was *Sulfurovum* sp. (phylum *Campilobacterota*), found mostly in the deep sediments (sampled at station 6841), where its abundance increased with depth up to 4.2% in the 16–19 cm horizon. *Sulfurovum sp.* can use oxygen and nitrate as electron acceptors and gain energy by oxidizing reduced sulfur compounds through the sulfur-oxidizing (Sox) pathway [90,91]. Since *Sulfurovum* was found in the anoxic zone, the most likely electron acceptor is nitrate, which is reduced to nitrogen gas. In oxygen minimum zones of the marine ecosystems, nitrate is the preferred alternative electron acceptor, and its reduction to gaseous N_2_O or N_2_ leads to loss of nitrogen to the atmosphere [92,93].

Thus, the composition of microbial communities indicates that in the anoxic zone of sediments, a full sulfur cycle can occur, including the reduction of sulfate to sulfide and the nitrate-dependent reverse oxidation of reduced sulfur compounds to sulfate.

### 4.3. Nitrogen Cycle

Nitrate is available from the seawater, where its concentration in the near-bottom horizons is within the micromolar range [94] and where it could be produced from ammonia in the nitrification process. The first step of this process, oxidation of ammonia to nitrite, could be performed both by bacteria and archaea. *Crenarchaeota* of the family *Nitrosopumilaceae* play a key role in the oxidation of ammonia to nitrite in marine ecosystems and global nitrogen cycles [48]. *Nitrosopumilaceae* were highly abundant (up to 27.6%) in the upper layers of the sediments, consistent with their aerobic lifestyle. Among ammonia-oxidizing bacteria, the upper levels of sediments harbored gamma-proteobacteria of the family *Nitrosococcaceae* (up to 1.7%) and the genus *Nitrosomonas* (up to 1.1%) [95,96]. 

The subsequent step of oxidation of nitrite to nitrate could be performed by members of the genera *Nitrospira* (phylum *Nitrospirota*) and *Nitrospina* (phylum *Nitrospinota*). They were found in all samples of upper sediments but in small amounts (up to 0.6% and 0.4%, respectively). Some *Nitrospira* can perform complete oxidation of ammonia to nitrate via nitrite, known as the commamox process [97]. Some strains of *Nitrospira* can perform the reverse process of nitrate reduction using H_2_ or formate as an electron donors and can exploit these energy sources concurrently with aerobic nitrite oxidation [98]. Nitrate-reducing SOB, as well as various heterotrophic nitrate and nitrite reducers, could complete the nitrogen cycle, generating ammonia and nitrogen gas.

### 4.4. Organic Matter Decomposition in Anoxic Sediments

Several lineages of bacteria and archaea were abundant in deep sediments at station 6841 (horizons 6–7 cm and 16–19 cm) but were absent or made up a much smaller fraction of communities in oxic surface sediments at all stations. In addition to the aforementioned ANME archaea, *Campilobacterota* and *Desulfobacterota*, such distribution pattern was observed for Marine Benthic Group D (MBG-D) archaea and bacterial phyla *Aminicenantes* (recognized as class *Aminicenantia* of the phylum *Acidobacteriota* in the GTDB taxonomy), *Caldatribacteriota* (JS1 lineage), and *Chloroflexi*.

MBG-D is an ecologically important uncultured archaeal lineage frequently found in anoxic marine sediments [99]. Metabolic analysis of metagenome-assembled genomes (MAGs) indicated that MBG-D archaea can utilize proteinaceous substances by fermentation, generating acetate and ethanol [99]. In addition, the MBG-D genomes might encode two autotrophic carbon fixation pathways: the Wood–Ljungdahl (WL) pathway and a modified dicarboxylate/4-hydroxybutyrate cycle and therefore has mixotrophic metabolism [100].

Anaerobic destruction of organic matter in the deep layers of sediments may be also performed by *Aminicenantes*. This uncultured bacterial lineage was detected by molecular methods in various terrestrial and marine ecosystems, mostly with low oxygen content [101], and comprises organisms with diverse metabolic capabilities, including fermentation of carbohydrates and/or proteinaceous substrates, acetate oxidation via the Wood–Ljungdahl pathway, and anaerobic respiration [102,103].

The JS1 lineage is one of the most abundant bacterial groups in anoxic marine sediments, especially in organic-rich or gas hydrate-containing sites [104]. Metabolic reconstruction of JS1 MAGs suggested that these bacteria are heterotrophic anaerobes that can perform fermentation of carbohydrates and syntrophic acetate oxidation [104,105].

*Chloroflexi* of the classes *Dehalococcoidia* and *Anaerolineae* were most abundant in the deep sediments at station 6841, where they accounted for almost one half of 16S rRNA gene sequences but also occurred in the upper levels. As in other studies, the detected *Chloroflexi* members were only distantly related to cultivated species of organohalide-respiring *Dehalococcoidia* [106,107] and organoheterotrophic *Anaerolineae* [108]. MAG-based analysis of the metabolic potential of *Chloroflexi* from subseafloor habitats revealed the ubiquitous presence of the Wood–Ljungdahl pathway, along with degradation pathways for complex carbohydrates, detrital proteins, aromatic compounds, and hydrogen, indicating the coupling of oxidation of diverse organics to CO_2_ reduction [109]. These bacteria can act as primary fermenters and acetogens without the need for syntrophic H_2_ consumption [109]. Interestingly, the *Dehalococcoidia* genomes contained reductive dehalogenase genes [109], suggesting that organohalide respiration is an important energy-yielding pathway in the subseafloor ecosystems [110].

Overall, lineages specific for the deep sediments mostly appeared to be anaerobic heterotrophs capable of fermentation of carbohydrates, fatty acids, and proteinaceous substrates, with concomitant production of hydrogen and low molecular weight organics that could be further mineralized by sulfate- and nitrate-respiring bacteria.

### 4.5. Microbial Processes of Transformation of Organic Matter in Sediments of the Arctic Seas: The Role of Methane

Bottom sediments of the Arctic seas are very heterogeneous in organic matter content, but they are even more heterogeneous in methane content (from 0.01 to more than 10^3^ µmol L^−1^). The source of methane in sediments is both modern organic matter, processed by the microbial community, and deep methane deposits such as gas hydrates. Not only the availability of organic matter but also the methane content determines the composition of microbial communities, as well as the rates of microbial biogeochemical processes. Unfortunately, with a fairly large number of studies devoted to the molecular analysis of microbial communities, there are only a few publications that present data on the rates of microbial processes in bottom sediments in the Arctic Ocean. Table 4 summarizes data on the rates of microbial processes of transformation of organic matter, oxidation of methane, sulfate reduction, and physicochemical characteristics of surface sediments (<50 cm depth) of various Arctic seas. In the sediments of the regions of the Laptev Seas, distant from the mouths of large Siberian rivers and outside of methane seep areas, the rates of microbial processes are very low, reducing conditions are rare, and the alkalinity is close to that of seawater. Such sediments, originating mainly from autochthonous-suspended organic matter, products of shore erosion, and eolian matter, are most typical for the Arctic Ocean. On the contrary, the rates of microbial biogeochemical processes increases manifold in sediments in sites of methane release such as mud volcanoes and methane seeps. The composition of microbial communities of sediments in places where methane is released to the seafloor is also very different from background areas [21,22,23,24].

Our results show that methane concentrations and rates of microbial processes in sediments in the northern part of the Barents Sea were noticeably higher than in oligotrophic areas of the Laptev Sea, but they were orders of magnitude lower than in the methane seep areas in the Laptev Sea and in the Haakon Mosby Mud Volcano zone (Table 4). The observed values of methane concentrations in the sediments of the Barents Sea, as well as the rates of biogeochemical processes, turned out to be the closest to the corresponding values of the southwestern part of the Kara Sea, which is a part of the West Siberian oil- and gas-bearing province. Thus, an increase in methane concentration, due to methane flow from deep sediments caused by destruction of oil and gas accumulations or a higher rate of organic matter sedimentation, significantly activates microbial processes in the bottom sediments.

## 5. Conclusions

**Figure 5:**
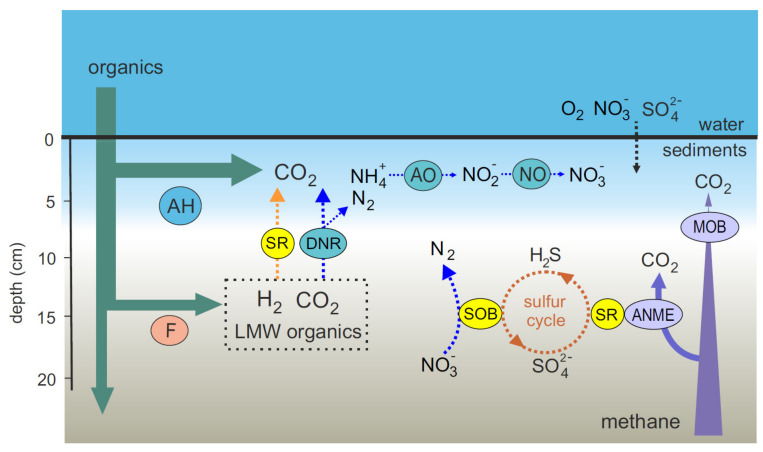
Microbial processes related to methane, sulfur, and nitrogen cycling in the Barents Sea sediments. AH, aerobic heterotrophs; F, fermentative microorganisms; SOB, sulfur-oxidizing bacteria; SR, sulfate-reducing bacteria; ANME, anaerobic methane-oxidizing archaea; MOB, aerobic methane-oxidizing bacteria; AO, ammonia-oxidizing microorganisms; NO, nitrite-oxidizing microorganisms; DNR, dissimilatory nitrate reducers; LMW, low molecular weight.

Although sampling of the deep layers of sediments was carried out only at one of the five stations, our data revealed differences between microbial communities and processes in the upper and deep sediments, probably reflecting oxic versus anoxic conditions (Figure 5). The upper layer was dominated by autotrophic ammonium-oxidizing *Crenarchaeota* and various groups of typical aquatic aerobic heterotrophic bacteria of the phyla *Actinobacteria, Proteobacteria, Verrucomicrobia*, and *Bacteroidetes* fed by falling organics. In the deep sediments, the sulfur and nitrogen cycles seemed to be linked. Nitrate formed as a result of ammonia oxidation is utilized by *Campilobacterota*, which oxidize sulfide formed by sulfate reducers back to sulfate. Nitrate, in turn, is reduced to gaseous nitrogen, and possibly to ammonia. Methane arriving from sediment layers located below the sulfate-rich zone is oxidized by ANME archaea in the anoxic zone in a process coupled to sulfate reduction and denitrification, or by aerobic methanotropic bacteria in the upper oxygenated layer. Methane concentrations and rates of microbial biogeochemical processes in sediments in the northern part of the Barents Sea are noticeably higher than in oligotrophic areas of the Arctic Ocean, indicating that an increase in methane concentration significantly activates microbial processes in the sediments.

## Figures and Tables

**Table 1:** The sampling stations.

Sampling Station	Sea Depth (m)	Coordinates	Sediment Depth (cm)	16S rRNA Gene Sequences *
6840	1514	75.21990 N 13.11843 E	0–1	10,140
6841	385	76.06437 N 15.57961 E	0–5	19,649
			6–7	16,146
			16–19	17,395
6844	101	77.03582 N 25.58852 E	0–1	174,986
6849	307	78.59960 N 35.39939 E	0–1	23,286
6864	584	80.59010 N 40.45922 E	0–1	22,583

* merged read pairs.

**Table 2:** Chemistry and microbial processes in the sediment samples.

Station	Sediment Depth, cm	Eh (mV)	Alk (mM)	Methane (μM)	MO (nmol L^−1^ day^−1^)	Sulfate (mM)	SR (µmol L^−1^ day^−1^)	DCA (µmol L^−1^ day^−1^)	TMC (×10^6^ cells ml^−1^)
6840	0–1	+100	2.4	0.288	6.2 ± 0.28	28.0	1.73 ± 0.16	2.71 ± 0.22	1.40 ± 0.12
6844	0–1	+125	2.2	0.597	8.2 ± 0.40	27.3	2.16 ± 0.21	5.75 ± 0.46	1.65 ± 0.17
6849	0–1	+110	2.4	0.217	6.6 ± 0.29	27.6	1.12 ± 0.12	4.12 ± 0.29	1.30 ± 0.15
6864	0–1	+110	2.3	0.398	2.1 ± 0.7	27.9	0.27 ± 0.03	2.14 ± 0.17	0.90 ± 0.1
6841	0–5	+10	2.6	2.39	22.8 ± 0.95	27.1	2.77 ± 0.24	12.42 ± 1.1	1.96 ± 0.21
	6–7	−80	4.0	5.08	0.9 ± 0.05	26.5	2.00 ± 0.23	0.51 ± 0.04	0.60 ± 0.1
	16–19	−120	4.4	9.51	0.3 ± 0.03	26.0	0.97 ± 0.10	0.31 ± 0.03	0.40 ± 0.1

Abbreviations: Alk, alkalinity; MO, methane oxidation; SR, sulfate reduction; DCA, dark CO_2_ assimilation; TMC, total microbial count.

**Table 3:** Diversity of Archaea and Bacteria.

Station	Sediment Depth, cm	Chao1		Shannon	
		Bacteria	Archaea	Bacteria	Archaea
6840	0–1	643.8	20	4.92	1.91
6844	0–1	929.3	67.1	5.37	2.96
6849	0–1	994.9	31.5	5.62	1.81
6864	0–1	1097.9	66.1	5.6	2.52
6841	0–5	1241.2	63.3	5.94	1.78
6841	6–7	897.9	149.1	5.43	4.08
6841	16–19	561.8	128.4	4.43	2.96

Chao1, Chao 1 diversity index; Shannon, Shannon diversity index.

**Table 4:** Chemistry and microbial processes in sediments of the Arctic sea.

Site (Number of Samples)	Eh (mV)	Alkalinity (mM)	Methane (μM)	MO (nmol L^−1^ day^−1^)	SR (µmol L^−1^ day^−1^)	DCA (µmol L^−1^ day^−1^)	Reference
Northern part of the Kara Sea (2)	ND	ND	0.02 ÷ 0.3	2.2 ÷ 12	0.4 ÷ 4.2	ND	[111]
Southwestern part of the Kara Sea (18)	−160 ÷ +40	2.4 ÷ 8.0	1.9 ÷ 20.3	9.1 ÷ 103	0.46 ÷ 2.2	2.1 ÷ 11.8	[111]
Laptev Sea, outside the methane seep field (7)	+40 ÷ +180	2.2 ÷ 2.8	<0.012	<2	<0.05	0.01 ÷ 0.12	[46]
Laptev Sea, methane seep field (5)	−160 ÷ +20	5.5 ÷ 18.0	19 ÷ 539	460 ÷ 3900	0.34 ÷ 4.8	0.2 ÷ 40.4	[46]
Laptev Sea, near the Lena river delta (19)	ND	ND	0.4 ÷ 5.4	<5.7	ND	ND	[112]
HMMV (18)	−350 ÷ −200	15.0 ÷ 30.5	> 2000	1500 ÷ 70000	0.5 ÷ 394	1.5 ÷ 154	[20,25]
Northern part of the Barents Sea (7)	−120 ÷ +125	2.2 ÷ 4.4	0.2 ÷ 9.5	0.3 ÷ 23	0.3 ÷ 2.8	0.3 ÷ 12.4	this work

Abbreviations: HMMV, Haakon Mosby Mud Volcano; ND, not determined.

## Data Availability

The obtained 16S rRNA gene sequences were deposited in the NCBI Sequence Read Archive (SRA) and are available via the BioProject PRJNA737614. Sequences of *pmoA* OTUs have been deposited in GenBank under accession numbers MZ505100 (OTU9) and MZ505101 (OTU32).

## Supplementary Materials

The following are available online at https://www.mdpi.com/article/10.3390/microorganisms9112362/s1, Table S1: Relative abundance and taxonomic classification of OTUs.
